# Structure–Function Analysis of Individual Reversion Variants of the Engineered Mpp46Aa1 (PS2Aa1) N65 Protein

**DOI:** 10.3390/cancers18182980

**Published:** 2026-09-15

**Authors:** Natalia A. Bravo-Granados, Nohora Juliana Rueda-Forero, Lydia Visser, Miguel O. Suárez-Barrera

**Affiliations:** 1 Instituto de investigacion Masira, Facultad de Ciencias Médicas y de la Salud, Universidad de Santander, Bucaramanga 68001, Colombia; 2 Department of Pathology and Medical Biology, University Medical Center Groningen, University of Groningen, 9713 GZ Groningen, The Netherlands

**Keywords:** *Bacillus thuringiensis*, Mpp46Aa1 (PS2Aa1), site-directed mutagenesis, colorectal cancer, protein engineering

## Abstract

Background: Mpp46Aa1 (formerly PS2Aa1) is a β-pore-forming protein produced by Bacillus thuringiensis that exhibits selective cytotoxicity against cancer cells. Protein engineering has generated variants with improved antitumor activity, including the N65 variant, which contains three amino acid substitutions. Objective: In this study, three individual reversion variants of N65 were generated by site-directed mutagenesis to determine the contribution of each substitution to cytotoxicity, selectivity, and cell death mechanisms in the colorectal cancer cell lines SW480 and SW620 and the non-tumorigenic colonic epithelial cell line NCM460. Methods: Recombinant proteins were purified and evaluated using AlamarBlue viability assays, Annexin V/Cy3 staining, caspase-3/7 and caspase-9 activation assays, and JC-1 mitochondrial membrane potential analysis. Results: The reversion variants differentially affected the biological activity of N65. Among them, the D34N variant exhibited low IC_50_ values against both colorectal cancer cell lines, while maintaining selectivity towards non-cancerous cells. D34N also induced phosphatidylserine externalization, activation of caspase-3/7 and caspase-9, and mitochondrial membrane depolarization, indicating activation of the intrinsic apoptotic pathway. Structural analyses revealed that the reverted residues modified local interaction networks, suggesting that these positions may influence CD59 recognition and contribute to cellular selectivity. Conclusions: These findings demonstrate that the three substitutions present in N65 contribute unequally to its biological activity and identify residue 34 as a key determinant of Mpp46Aa1 cytotoxicity. This study provides structural and functional insights that support the rational engineering of parasporins with enhanced antitumor selectivity.

## Simple Summary

Colorectal cancer remains a major health challenge, highlighting the need for new treatments that can selectively target cancer cells while minimizing damage to healthy tissues. Mpp46Aa1 (PS2Aa1) is a protein produced by the bacterium Bacillus thuringiensis that has shown promising anticancer activity. This study investigated how three specific changes in the protein influence its ability to kill colorectal cancer cells. The researchers generated modified versions of the protein and evaluated their effects on cancer and non-cancerous cells, as well as the mechanisms involved in cell death. One variant, D34N, showed the strongest anticancer activity while maintaining selectivity toward cancer cells. It also activated processes associated with programmed cell death and affected mitochondrial function. These findings identify an important region of Mpp46Aa1 that may be useful for designing more selective and effective protein-based therapies against colorectal cancer.

## 1. Introduction

Cancer remains one of the leading causes of mortality worldwide. Among all malignancies, colorectal cancer (CRC) is the third most frequently diagnosed cancer and the second leading cause of cancer-related deaths, with approximately 2.04 million new cases and 917,895 deaths reported worldwide in 2024 [1]. CRC development is driven by the progressive accumulation of genetic and epigenetic alterations, resulting in extensive molecular heterogeneity that limits therapeutic efficacy [2]. Although advances in surgery, chemotherapy, targeted therapy, and immunotherapy have improved patient outcomes, treatment failure remains common because of drug resistance, systemic toxicity, and unequal access to advanced therapies [3]. Consequently, the identification of novel biological agents with high tumor selectivity and reduced toxicity has become an important research priority [4,5].

Parasporins are a family of non-insecticidal proteins produced by Bacillus thuringiensis that selectively kill cancer cells while exhibiting little or no toxicity toward normal mammalian cells [4,6]. Among them, PS2Aa1, currently designated Mpp46Aa1, belongs to the aerolysin-like β-pore-forming toxin family and has demonstrated selective cytotoxicity against several human cancer cell lines, including HepG2, Sawano, HL60, CaCo-2, Jurkat, and MOLT-4 [4,7,8]. Structurally, Mpp46Aa1 consists of three functional domains. Domain I mediates receptor recognition and membrane binding, whereas Domains II and III undergo the conformational rearrangements required for oligomerization and transmembrane pore formation [9,10]. Although the cellular receptor has not been experimentally confirmed, glycosylphosphatidylinositol-anchored proteins have been identified as essential co-receptors for parasporin activity [11], and molecular dynamics studies have suggested that CD59 may represent a favorable binding partner for Mpp46Aa1 [4,11].

Protein engineering has emerged as an effective strategy to improve the biological activity of parasporins. Site-directed mutagenesis of Mpp46Aa1 generated the N65 variant, which contains three amino acid substitutions: N92D in Domain I, K175R in Domain II, and S218G in Domain III [8]. Previous studies demonstrated that N65 exhibits enhanced cytotoxicity against SW480, SW620, CaCo-2, MOLT-4, and Jurkat cells compared with the wild-type protein, indicating that these substitutions contribute to improved antitumor activity [4,8,12]. However, the individual contribution of each mutation to cytotoxicity, selectivity, and the underlying molecular mechanism remains unknown.

To address this question, we generated individual reversion variants of N65 by site-directed mutagenesis and performed an integrated functional and structural characterization. Cytotoxicity, apoptotic signaling, and mitochondrial dysfunction were evaluated in colorectal cancer and non-tumorigenic colon cell lines, whereas structural analyses were conducted to determine how each reverted residue altered local molecular interactions. This approach allowed us to establish structure–function relationships for the N65 substitutions and to identify residues that govern the cytotoxic activity and selectivity of Mpp46Aa1. These findings provide a molecular framework for the rational engineering of parasporins with improved therapeutic potential against colorectal cancer.

## 2. Materials and Methods

### 2.1. Bacterial Strains, Plasmids, and Cloning

The N65 variant cloned into pET30a(+) was cultured in Luria–Bertani (LB) medium supplemented with kanamycin (25 mg/L) at 37 °C for 24 h. Plasmid DNA was extracted using the FavorPrep™ Plasmid DNA Extraction Mini Kit (Favorgen Biotech, PingTung, Taiwan) according to the manufacturer’s instructions. The coding sequence was subsequently subcloned into the pFN29A expression vector and transformed into chemically competent *Escherichia coli* JM109 cells. White colonies displaying regular morphology were selected by blue–white screening and cultured in LB broth containing ampicillin (25 mg/L) for molecular verification.

### 2.2. Cell Culture

The human colorectal cancer cell lines SW480 and SW620 and the non-tumorigenic human colonic epithelial cell line NCM460 were used in this study. Cells were cultured in Dulbecco’s Modified Eagle Medium (DMEM; Thermo Fisher Scientific, Lenexa, KS, USA) supplemented with 10% fetal bovine serum (FBS) and maintained at 37 °C in a humidified atmosphere containing 5% CO_2_. The cell lines SW480 (primary tumor) and SW620 (lymph node metastasis) of colorectal cancer derived from the same patient were selected. This origin allows the biological activity of parasporins to be compared in biologically related contexts, reducing part of the variability associated with comparing lines from different patients. NCM460 was selected as a non-tumorigenic human colonic mucosal epithelial model, allowing comparison with tumor cells from the same tissue context.

### 2.3. Site-Directed Mutagenesis

Individual reversion variants were generated from the N65 construct using the GeneArt™ Site-Directed Mutagenesis Plus Kit (Thermo Fisher Scientific) with three pairs of mutation-specific primers, following the manufacturer’s protocol. PCR products were transformed into chemically competent *E. coli* JM109 cells, and positive clones were confirmed by Sanger sequencing. Sequence analyses were performed using UGENE v53.1, sequencing quality was assessed with FastQC v0.12, and nucleotide sequences were translated using the ExPASy Translate tool (standard genetic code).

N65 contains the N92D, K175R, and S218G substitutions relative to the native full-length PS2Aa1/Mpp46Aa1 precursor. The individual reversion constructs were generated in the N65 background to restore one corresponding native residue while retaining the other two N65 substitutions. For structural comparison, translated sequences were aligned with chain A of PDB 2ZTB, which represents the processed protein. Because 2ZTB differs from the 338-amino-acid precursor sequence, equivalent residues use different numbering. Thus, the precursor substitutions N92D, K175R, and S218G correspond to positions 34, 117, and 160, respectively, in the 2ZTB-based sequence, and the reversion variants were designated D34N, R117K, and G160S. Sequencing confirmed the expected reversions, preservation of the reading frame, and absence of additional coding-region mutations.

### 2.4. Recombinant Protein Expression and Purification

Recombinant His-tagged proteins were purified using the MagneHis™ Protein Purification System (Promega, Madison, WI, USA) according to the manufacturer’s instructions. Soluble protein extracts were incubated with nickel-coated magnetic particles to capture His-tagged recombinant proteins. Protein concentration was determined using the Bradford assay, with bovine serum albumin (BSA) as the standard, and absorbance was measured at 595 nm in triplicate. Purified fractions were analyzed by SDS-PAGE to verify protein integrity and purity. Recombinant proteins were subsequently activated by proteinase K prior to functional assays.

### 2.5. Cell Viability Assay

SW480, SW620, and NCM460 cells were seeded into 96-well plates at a density of 8 × 10^3^ cells per well and allowed to adhere overnight. Cells were treated with activated recombinant Mpp46Aa1, N65, or the corresponding reversion variants at 1, 2, 4, 5, 10, and 20 μg/mL for 72 h at 37 °C and 5% CO_2_. Cell viability was assessed using the AlamarBlue^®^ assay after 4–6 h of incubation, according to the manufacturer’s instructions. Metabolic activity was calculated as:

%Metabolic activity = (Abs treated cells)/(Abs non treated cells) × 100

Half-maximal inhibitory concentrations (IC_50_) were determined by nonlinear regression using GraphPad Prism v11.0 (GraphPad Software, San Diego, CA, USA). The selectivity index (SI) was calculated as:

%SI = (IC50 NCM460)/(IC50 Cancer cell line)

### 2.6. Annexin V Apoptosis Assay

Apoptosis was evaluated using the Annexin V/Cy3 Apoptosis Detection Kit (Sigma-Aldrich, St. Louis, MO, USA). Cells were seeded at 2 × 10^4^ cells per well and incubated for 24 h under standard culture conditions. Cells were then treated with recombinant proteins (5 μg/mL) for an additional 24 h. Subsequently, 50 μL of Annexin V/Cy3 reagent was added according to the manufacturer’s instructions, and fluorescence was measured using a Varioskan™ LUX multimode plate reader (Thermo Fisher Scientific) at 485/520 nm (Ex/Em). Untreated cells served as the negative control, whereas 2.5% DMSO was included as an assay positive control for stress-associated induction of Annexin V/Cy3 fluorescence; DMSO was not used as a vehicle for the recombinant proteins. NCM460 cells were included as a non-tumorigenic normal-cell control to assess the selectivity of the recombinant proteins.

### 2.7. Mitochondrial Membrane Potential Assay

Changes in mitochondrial membrane potential (ΔΨm) were determined using the JC-1 fluorescent probe in black, clear-bottom 96-well plates. Cells (2 × 10^4^ cells per well) were incubated for 24 h, followed by treatment with 5 μg/mL of Mpp46Aa1, N65, or D34N for an additional 24 h. After treatment, the culture medium was removed and cells were incubated with 1 μM JC-1 for 1 h at 37 °C. Assay buffer was then added according to the manufacturer’s protocol. Fluorescence was recorded using a Varioskan™ LUX at 490/525 nm (green monomers) and 540/590 nm (red aggregates). Mitochondrial membrane potential was expressed as:

Ratio = (Red signal)/(Green signal)

(%)ΔΨm = ((Sample ratio)/(Negative control ratio)) × 100

### 2.8. Caspase-3/7 and Caspase-9 Activity

The activities of caspase-3/7 and caspase-9 were determined using Caspase-Glo^®^ 3/7 and Caspase-Glo^®^ 9 Assay Kits (Promega, Madison, WI, USA). Cells (2 × 10^4^ cells per well) were treated with 5 μg/mL of recombinant proteins for 24 h. Equal volumes of the corresponding luminogenic substrate were then added and incubated for 60 min at room temperature in the dark. Luminescence was measured using a Varioskan™ LUX multimode plate reader. No standard pharmacological apoptosis inducer was included and untreated cells were used to establish basal activity.

### 2.9. Comparative Structural Analysis

For the comparative structural analysis, the 2ZTB A-chain was used as a reference. The N65 and D34N models were generated with SWISS-MODEL and analyzed in PyMOL version 3.0.4 (Schrödinger, LLC, New York, NY, USA), where solvents, ligands, and non-protein elements were removed. The structures were overlaid using the align command to obtain the RMSD values. The microenvironment of the residues of interest within a radius of 5 Å and the possible polar contacts with a 3.5 Å cut-off were evaluated. The electrostatic potential was calculated using PDB2PQR v3.6.1 and APBS v3.4.1 via the Poisson–Boltzmann web server and visualized with a common scale of −5 to +5 kT/e. No molecular dynamics simulations or independent energy minimization were performed, so the analysis corresponds to a static structural comparison.

### 2.10. Statistical Analysis

Each functional assay was performed in three independent biological experiments, with three technical replicates per condition within each experiment. Data were expressed as mean ± SD. Statistical differences among groups were analyzed using one-way analysis of variance (ANOVA) followed by Tukey’s multiple-comparison test. IC_50_ values were estimated by nonlinear regression using GraphPad Prism, and differences were considered statistically significant at *p* < 0.05.

## 3. Results

### 3.1. Generation and Molecular Validation of Individual Reversion Variants Derived from N65

**Figure 1:**
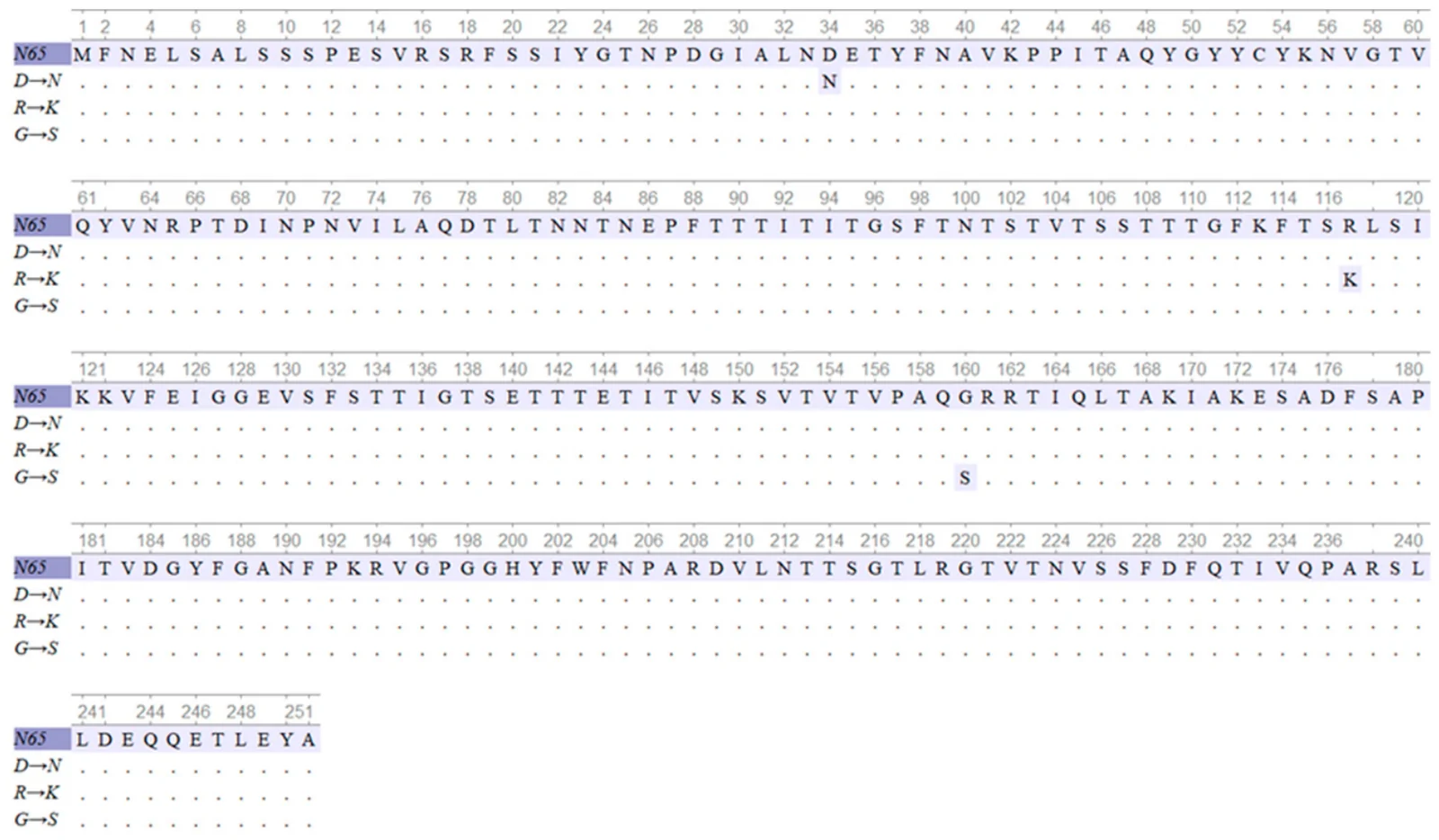
Amino acid sequence alignment generated using UGENE. The upper sequence corresponds to the parental N65 variant, whereas the lower sequences represent the individual reversion variants generated by site-directed mutagenesis: D34N, R117K, and G160S.

Site-directed mutagenesis was employed to individually restore the amino acid substitutions present in the N65 variant. Three independent reversion variants were successfully generated, corresponding to D34N, R117K, and G160S according to the processed PS2Aa1/Mpp46Aa1 sequence represented by chain A of PDB 2ZTB, and were confirmed by Sanger sequencing at Macrogen Inc. using PS2-specific primers previously described by Brasseur et al. (2015) [7]. The nucleotide sequences were analyzed and aligned in UGENE against the sequence corresponding to chain A of Mpp46Aa1 (PDB: 2ZTB), obtaining 99.18% sequence identity. Sanger sequencing confirmed the presence of the expected nucleotide substitutions and verified the absence of additional mutations within the coding region. Sequence alignment with the parental translated N65 variant (Figure 1) further validated the successful generation of all three reversion variants. These molecularly confirmed variants were subsequently used for functional and structural characterization.

Comparison of the amino acid sequences of the three variants with chain A of the PS2Aa1/Mpp46Aa1 crystal structure (PDB 2ZTB) showed 99.18% sequence identity. The apparent difference between the reversion designations and the substitutions originally reported for N65: N92D, K175R, and S218G results from the use of different numbering systems. N65 was originally described relative to the full-length 338-amino-acid precursor, whereas 2ZTB represents the proteolytically processed 252-amino-acid toxin. Thus, D34N, R117K, and G160S correspond, respectively, to individual reversions of the N65 substitutions N92D, K175R, and S218G in precursor numbering. Therefore, the amino acids are equivalent to the precursor nomenclature used by Alarcón-Aldana et al. (2024) [4].

In each construct, one N65-derived substitution was restored to the corresponding native PS2Aa1/Mpp46Aa1 residue, while the other two N65 substitutions were retained. These results confirm the molecular identity and specificity of the D34N, R117K, and G160S reversion variants, validating their suitability for subsequent functional and structural analyses aimed at defining the contribution of each residue to the cytotoxic phenotype of Mpp46Aa1 in colorectal cancer cells.

### 3.2. Recombinant Protein Expression and Purification

**Figure 2:**
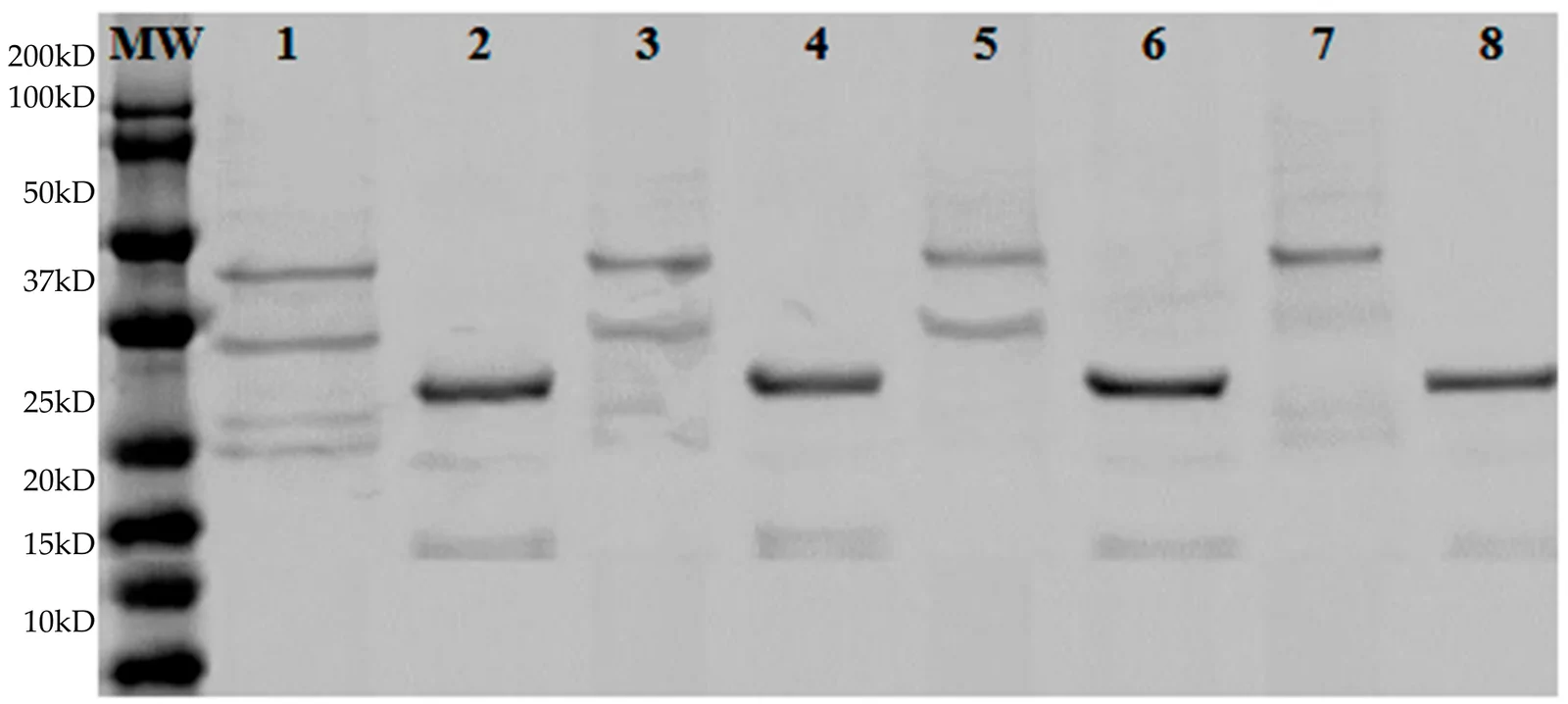
SDS-PAGE analysis of purified recombinant Mpp46Aa1 proteins. Bands of approximately 37 kDa and 30 kDa correspond to the precursor and activated forms, respectively. Lane 1: precursor N65; Lane 2: activated N65; Lane 3: precursor D34N; Lane 4: activated D34N; Lane 5: precursor R117K; Lane 6: activated R117K; Lane 7: precursor G160S; Lane 8: activated G160S. All proteins were purified using the MagneHis™ Protein Purification System. The purity of the protein preparations was assessed qualitatively by SDS-PAGE but was not quantified by densitometry. The uncropped blots are shown in Supplementary Materials.

Recombinant proteins were purified using the MagneHis™ Protein Purification System (Promega), which employs nickel-coated magnetic particles for the affinity purification of histidine-tagged proteins. Protein expression and purification were assessed by SDS-PAGE, revealing bands of approximately 37 kDa and 30 kDa, corresponding to the precursor and proteolytically activated forms of the proteins, respectively. These bands were consistently detected for the R117K, D34N, G160S, and N65 variants (Figure 2), confirming successful recombinant protein production and purification for downstream functional characterization.

Proteolytic activation with proteinase K generated a predominant protein band of approximately 30 kDa for all variants, indicating successful processing of the recombinant protoxins into their active forms. The observed reduction in molecular weight is consistent with the reported activation mechanism of Mpp46Aa1 and previous studies on parasporin-2. Faint secondary bands detected in the non-activated samples likely represent residual contaminants, partially processed intermediates, or minor purification by-products. Overall, these results confirm the efficient proteolytic activation and purification of the recombinant proteins prior to biological characterization.

### 3.3. Cytotoxic Activity of Wild-Type Mpp46Aa1, the N65 Variant, and Individual Reversion Variants

**Figure 3:**
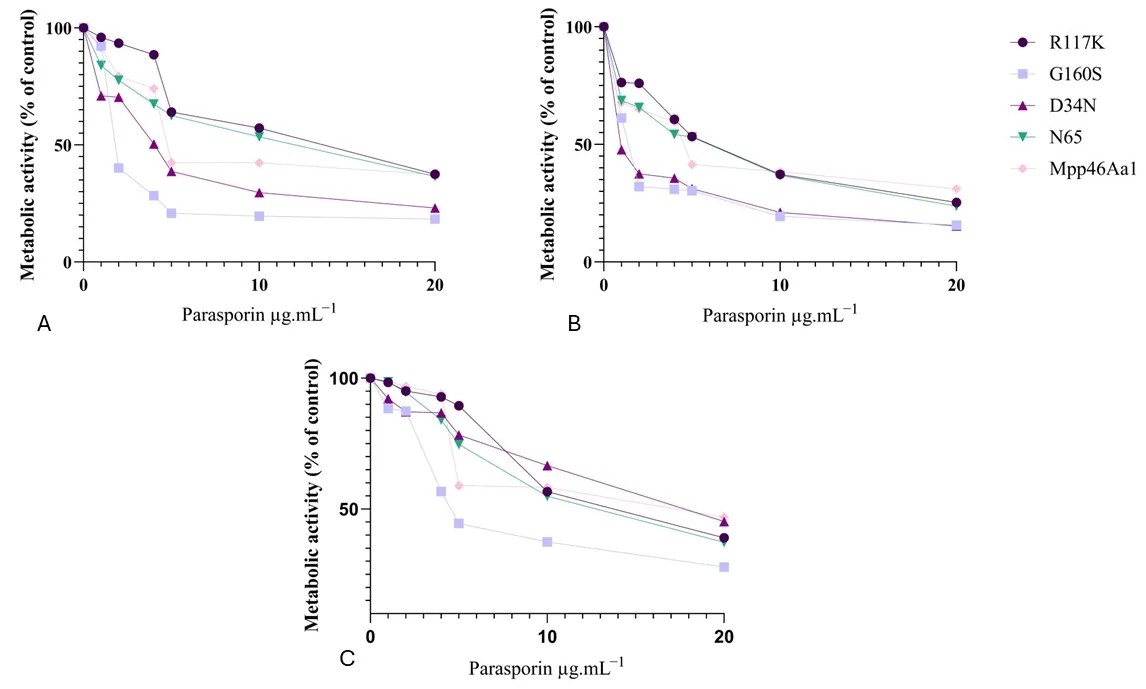
Dose-dependent effects of recombinant Mpp46Aa1 proteins on cell metabolic activity. (**A**) SW480 colorectal cancer cells, (**B**) SW620 metastatic colorectal cancer cells, and (**C**) non-tumorigenic NCM460 colonic epithelial cells. Cell viability was quantified after 72 h of treatment using the AlamarBlue fluorescence assay and is expressed as the percentage of relative metabolic activity normalized to untreated controls. Data are presented as mean ± SD of three replicates per treatment.

The cytotoxic activity of wild-type Mpp46Aa1, the N65 variant, and the individual reversion variants was evaluated in the colorectal cancer cell lines SW480 and SW620, and in the non-tumorigenic colonic epithelial cell line NCM460 following 72 h of treatment (Figure 3). Using a single non-tumor line limits generalization, and the observed selectivity should be described as selectivity relative to NCM460 in this experimental system, rather than definitive evidence of cancer-specificity.

Cell viability was determined using the AlamarBlue fluorescence assay after exposure to protein concentrations of 1, 2, 4, 5, 10, and 20 μg/mL. Results are expressed as the percentage of relative metabolic activity normalized to untreated controls. This analysis enabled comparison of the sensitivity of each cell line to the different Mpp46Aa1 variants and established the basis for assessing the contribution of individual amino acid substitutions to cytotoxic activity and cellular selectivity.

To quantitatively compare the cytotoxic effects of the recombinant proteins, dose–response curves were fitted to estimate the half-maximal inhibitory concentration (IC_50_) for each variant in the three cell lines. These values enabled a direct comparison of cytotoxic potency and cellular selectivity among wild-type Mpp46Aa1, the N65 variant, and the individual reversion variants (Table 1).

The G160S reversion variant exhibited the lowest IC_50_ values in both SW480 (2.14 μg/mL) and SW620 (1.57 μg/mL) cells, indicating the highest cytotoxic potency among the evaluated proteins. However, it also displayed a low IC_50_ value in NCM460 cells, reflecting limited discrimination between malignant and non-malignant cells and, consequently, poor tumor selectivity. In contrast, the D34N variant required slightly higher concentrations to reduce the metabolic activity of SW480 (3.86 μg/mL) and SW620 (1.52 μg/mL) cells but exhibited markedly lower cytotoxicity toward NCM460 cells, suggesting an improved therapeutic profile. These findings indicate that cytotoxic potency alone does not predict the suitability of a protein as an anticancer candidate, highlighting the importance of evaluating cellular selectivity.

To further assess the therapeutic potential of the recombinant proteins, the selectivity index (SI) was calculated by comparing the IC_50_ values obtained in non-tumorigenic and colorectal cancer cells. The SI provides a quantitative measure of preferential toxicity toward cancer cells and represents a key parameter for the preclinical evaluation of candidate anticancer proteins. The calculated SI values are summarized in Table 2.

Selectivity index (SI) values >1 indicate preferential sensitivity of cancer cells to the recombinant proteins, whereas SI ≥ 2 is generally considered indicative of acceptable biological selectivity. Conversely, SI < 1 reflects greater toxicity toward non-tumorigenic cells than toward cancer cells [13,14]. Based on these criteria, the G160S (SI = 2.83), D34N (SI = 4.75), and wild-type Mpp46Aa1 (SI = 2.12) proteins exhibited favorable selectivity toward SW480 cells relative to NCM460 cells. In SW620 cells, all recombinant proteins displayed SI values > 2, indicating acceptable tumor selectivity across all variants evaluated.

D34N was selected for subsequent mechanistic analyses due to its favorable profile against both colorectal cancer lines, with SI values of 4.75 for SW480 and 12.06 for SW620. Although G160S showed high cytotoxic activity, it also had a greater effect on NCM460, which reduced its selectivity, while R117K exhibited a lower selective profile.

Among the proteins tested, the D34N variant demonstrated the most favorable balance between cytotoxic potency and tumor selectivity, particularly against the metastatic SW620 cell line. Its enhanced cytotoxic activity toward colorectal cancer cells, together with its reduced toxicity toward non-tumorigenic cells, identified D34N as the most promising candidate for mechanistic characterization. Accordingly, this variant was selected for subsequent studies investigating the cellular mechanisms underlying Mpp46Aa1-induced cell death.

### 3.4. Functional Characterization of the Cytotoxic Activity of D34N, N65, and Wild-Type Mpp46Aa1

To investigate the mechanisms responsible for the observed cytotoxic effects, complementary assays were performed to evaluate plasma membrane alterations, apoptosis, and mitochondrial dysfunction. A protein concentration of 5 μg/mL was used in all experiments to ensure standardized experimental conditions and facilitate direct comparison among treatments.

**Figure 4:**
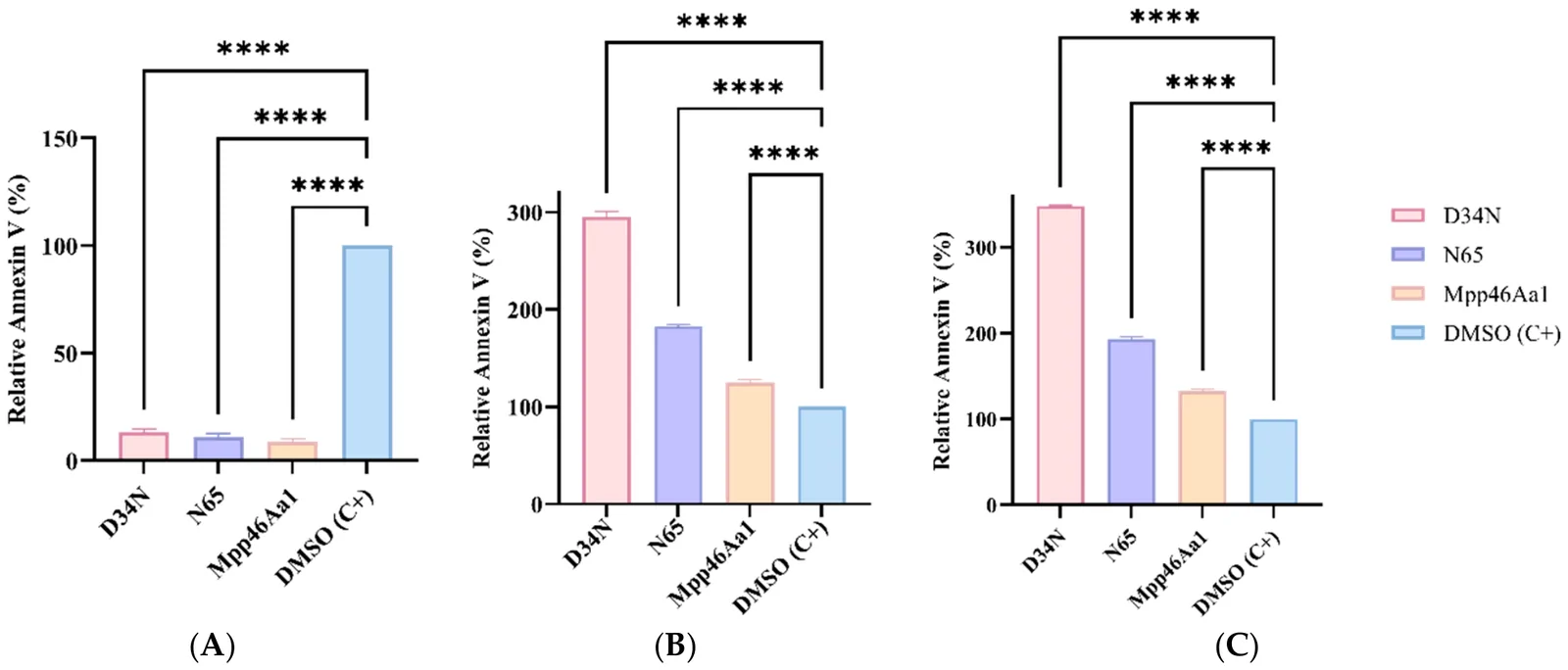
Assessment of apoptosis by Annexin V/Cy3 staining in (**A**) NCM460, (**B**) SW480, and (**C**) SW620 cells after 24 h treatment with wild-type Mpp46Aa1, N65, or D34N proteins (5 μg/mL). Untreated cells served as the negative control. Cells treated with 2.5% DMSO served as the assay positive control. Apoptosis was quantified by measuring Annexin V/Cy3 fluorescence as an indicator of phosphatidylserine externalization. Statistical significance between the positive control and treated cells was determined using one-way ANOVA (**** *p* < 0.0001). Parasporin (5 µg·mL^−1^). Data are presented as mean ± SD from three independent biological experiments (*n* = 3), each performed with three technical replicates.

The apoptotic response induced by wild-type Mpp46Aa1, the N65 variant, and the D34N reversion variant was initially assessed by Annexin V/Cy3 staining (Figure 4), which enabled the detection of phosphatidylserine externalization as an early marker of apoptosis in the evaluated cell lines.

In the non-tumorigenic NCM460 cell line, Annexin V positivity remained low following treatment with D34N, N65, and wild-type Mpp46Aa1, ranging from 8% to 13%, indicating minimal phosphatidylserine externalization under the experimental conditions, while DMSO induced a clear increase in Annexin V/Cy3 signal in NCM460 cells. The 2.5% DMSO positive control produced a strong Annexin V signal, confirming the functionality of the assay under the experimental conditions used. In contrast, both colorectal cancer cell lines exhibited a marked increase in Annexin V staining, consistent with apoptosis induction. The D34N variant produced the strongest response, with relative Annexin V signals of 295.3% in SW480 cells and 348.0% in SW620 cells, followed by N65 (182.6% and 193.0%, respectively) and wild-type Mpp46Aa1 (125.0% and 132.6%, respectively). Compared with the 2.5% DMSO control, all treatments induced statistically significant increases in Annexin V fluorescence (*p* < 0.0001), demonstrating that recombinant Mpp46Aa1 proteins selectively promoted phosphatidylserine externalization in colorectal cancer cells while exerting minimal effects on non-tumorigenic cells.

**Figure 5:**
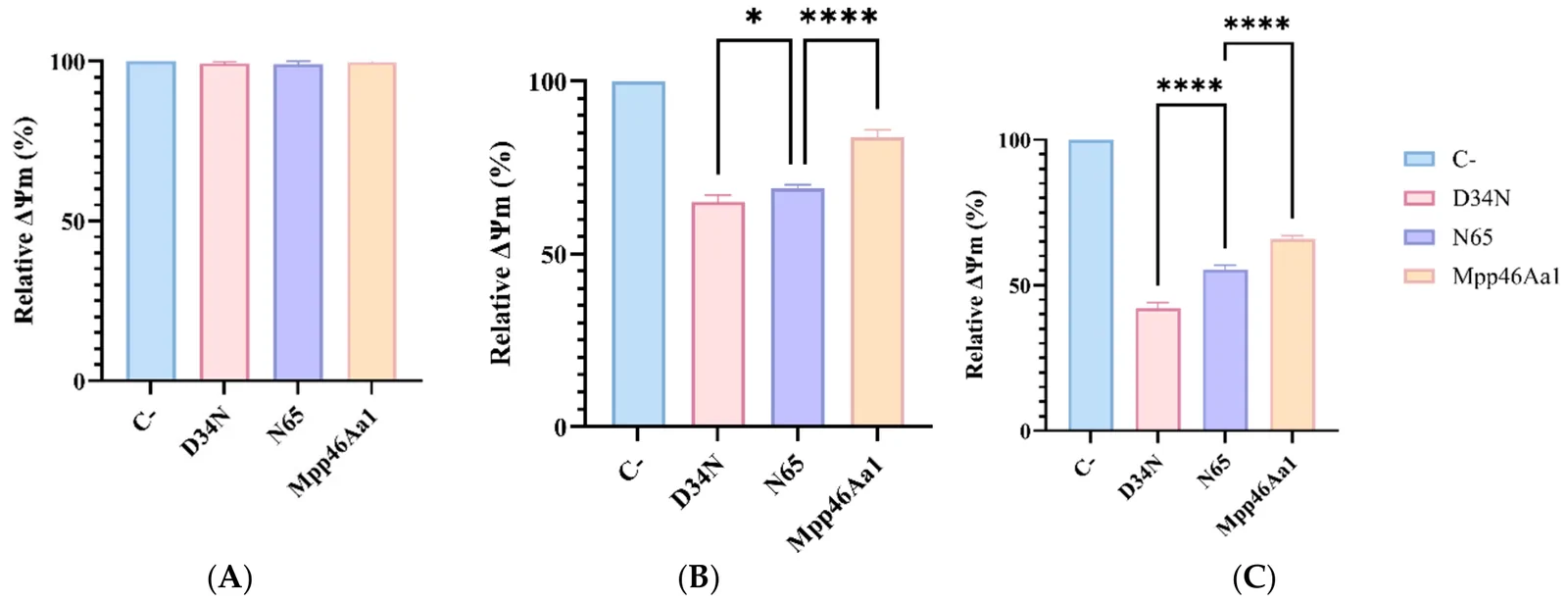
Evaluation of mitochondrial membrane potential (ΔΨm) using the JC-1 assay in (**A**) NCM460, (**B**) SW480, and (**C**) SW620 cells following treatment with recombinant Mpp46Aa1 proteins (5 μg/mL, 24 h). Results are expressed as the relative mitochondrial membrane potential normalized to the untreated control (C−). Statistical significance between untreated and treated groups was determined by one-way ANOVA (* *p* < 0.05 and **** *p* < 0.0001). Parasporin (5 µg·mL^−1^). Data are presented as mean ± SD from three independent biological experiments (*n* = 3), each performed with three technical replicates.

To further investigate the mechanism of cell death, mitochondrial membrane potential (ΔΨm) was evaluated using the JC-1 assay. Relative ΔΨm values obtained from SW480, SW620, and NCM460 cells following treatment with recombinant proteins (5 μg/mL, 24 h) are presented in Figure 5. This analysis was performed to determine whether mitochondrial depolarization contributes to the cytotoxic activity of Mpp46Aa1 and its engineered variants.

Following treatment, a marked reduction in the JC-1 aggregate-to-monomer ratio was observed in both SW480 and SW620 colorectal cancer cells, indicating mitochondrial membrane depolarization. Among the proteins evaluated, the D34N variant induced the greatest loss of mitochondrial membrane potential, particularly in SW620 cells. This pronounced depolarization consists of mitochondrial dysfunction and supports the involvement of the intrinsic apoptotic pathway, in which disruption of ΔΨm represents an early event preceding caspase activation.

**Figure 6:**
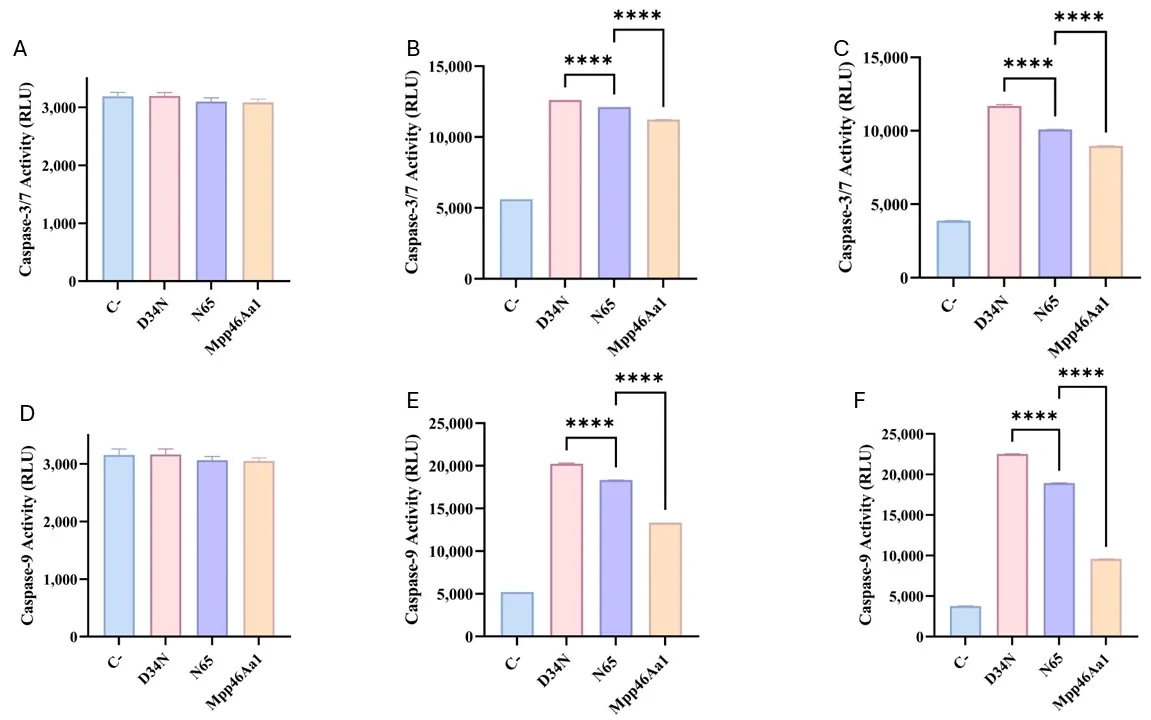
Caspase-3/7 and caspase-9 activities following treatment with recombinant Mpp46Aa1 proteins. Luminescence was recorded as relative luminescence units (RLU). Values obtained from three independent biological experiments were averaged and results are expressed as mean ± SD (*n* = 3). (**A**,**D**) NCM460, (**B**,**E**) SW480, and (**C**,**F**) SW620 cells. Statistical significance was determined using one-way ANOVA (**** *p* < 0.0001). Parasporin (5 µg·mL^−1^).

To determine whether mitochondrial depolarization was associated with activation of the intrinsic apoptotic cascade, the activities of initiator caspase-9 and effector caspases-3/7 were subsequently quantified (Figure 6, Supplemental Table S1). These analyses were performed to establish the relationship between mitochondrial dysfunction and apoptotic signaling induced by wild-type Mpp46Aa1, the N65 variant, and the D34N reversion variant.

The combined results from the Annexin V, JC-1, and caspase assays indicate that the recombinant Mpp46Aa1 proteins preferentially induce apoptosis in the colorectal cancer cell lines SW480 and SW620, while producing only limited effects in the non-tumorigenic NCM460 cells. The loss of mitochondrial membrane potential, together with the marked activation of caspase-9, supports the involvement of the intrinsic (mitochondrial) apoptotic pathway. Furthermore, the increased activity of caspases-3/7 demonstrates progression toward the execution phase of apoptosis.

Among the proteins evaluated, the D34N variant consistently showed the strongest apoptotic response, particularly in the metastatic SW620 cell line, suggesting enhanced activity against cells with a more aggressive phenotype. Although these findings support the improved antitumor potential of D34N, the molecular basis underlying its enhanced activity remains to be elucidated. Additional studies focused on receptor recognition, membrane interactions, and apoptosis-related molecular markers will be required to define the mechanisms responsible for its increased cytotoxicity and selectivity.

### 3.5. Comparative Structural Analysis

**Figure 7:**
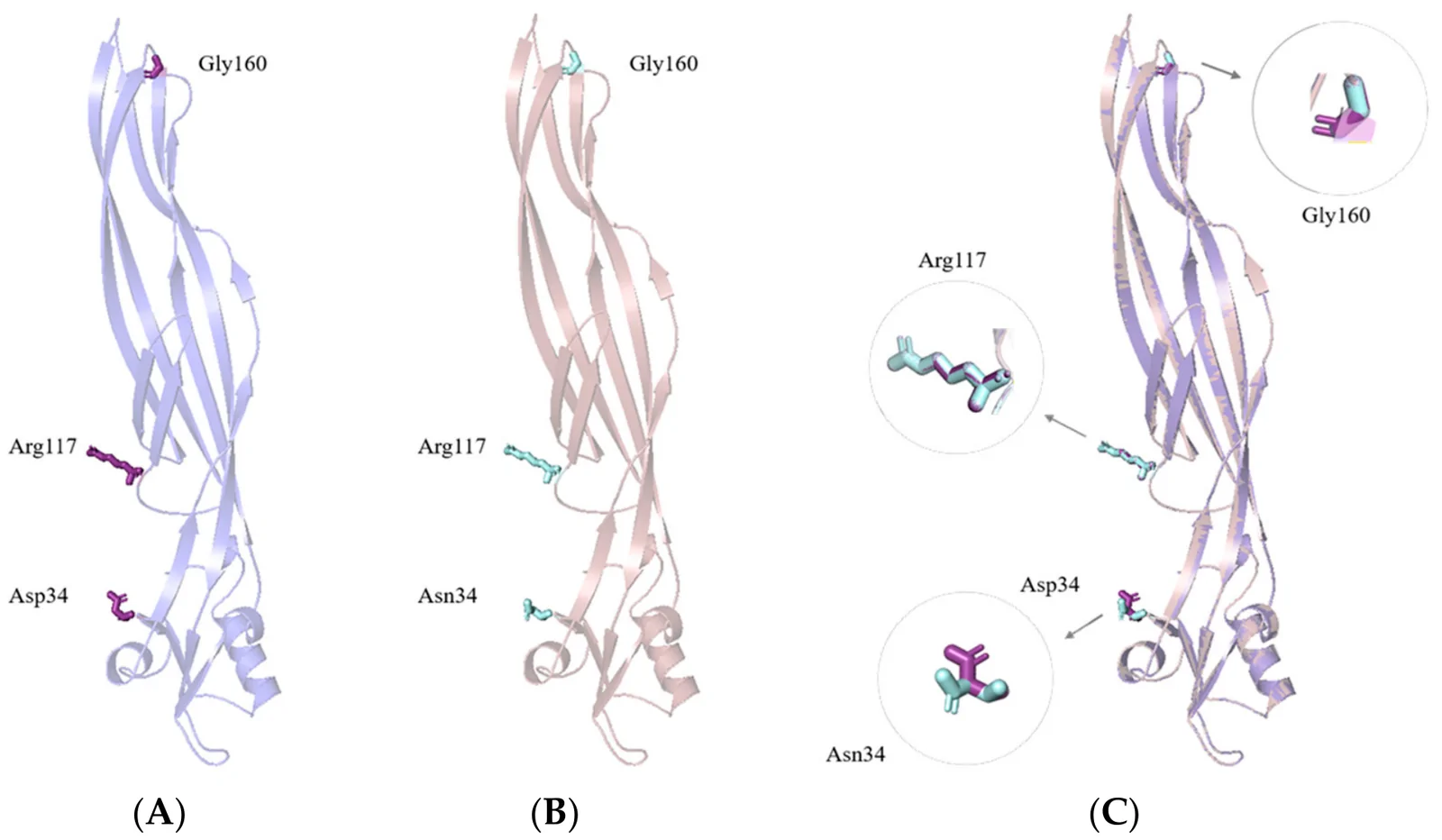
Structural superposition of Mpp46Aa1 variants. (**A**) N65 variant. (**B**) D34N reversion variant. (**C**) Structural superposition of N65 and D34N. Residues corresponding to the evaluated amino acid substitutions are highlighted to illustrate their spatial localization within the protein structure.

To establish the structural basis for the functional differences observed among the recombinant proteins, a comparative structural analysis was performed. Structural superposition of the N65 variant and the D34N reversion variant is shown in Figure 7, while the corresponding root mean square deviation (RMSD) values for each structural comparison are summarized in Table 3. This analysis was conducted to determine whether local structural changes associated with the reverted residues could explain the differences in cytotoxic activity and cellular selectivity.

Structural superposition of the N65 and D34N variants revealed a high degree of conservation of the overall protein fold. Low RMSD values were obtained for all structural comparisons, including 0.002 Å for both N65 and D34N relative to the reference crystal structure (2ZTB) and 0.001 Å for the comparison between D34N and N65. These results demonstrate that both variants closely overlap with the reference structure, indicating that the amino acid substitutions present in N65 and the D34N reversion do not induce detectable global conformational changes.

The preservation of the overall protein architecture suggests that the functional differences observed among the variants are more likely attributable to local structural perturbations rather than large-scale conformational rearrangements. Such localized changes may influence molecular recognition, membrane interactions, or receptor binding, thereby modulating the cytotoxic activity of Mpp46Aa1 against colorectal cancer cells.

To further investigate these local effects, electrostatic surface potential analysis was performed to evaluate changes in the physicochemical properties surrounding the substituted residues.

Local electrostatic surface potential analysis revealed discrete changes in the regions surrounding the substituted residues, with the most pronounced differences observed around residue 34. The Asp → Asn reversion in the D34N variant replaces a negatively charged acidic residue with an uncharged polar residue, resulting in local neutralization of the electrostatic surface. This modification is expected to alter the local physicochemical environment and may influence electrostatic complementarity with membrane components or putative cellular receptors, including the proposed interaction with CD59.

Importantly, these local electrostatic changes occurred without detectable alterations in the global three-dimensional structure of the protein, supporting the hypothesis that subtle local physicochemical modifications, rather than large conformational rearrangements, underlie the enhanced cytotoxic profile of the D34N variant. Although these observations provide a plausible structural explanation for the functional differences among the variants, they should be interpreted as evidence from comparative structural modeling and do not demonstrate protein dynamics or the molecular mechanism of receptor recognition.

Taken together, the structural and functional findings identify D34N as the most promising engineered Mpp46Aa1 variant and provide a mechanistic framework for the rational design of parasporins with improved tumor selectivity and anticancer activity. These results establish a foundation for future studies integrating structural biology, receptor-binding analyses, and molecular dynamics simulations to further elucidate the determinants of Mpp46Aa1 specificity and function.

## 4. Discussion

Parasporins are proteins produced by Bacillus thuringiensis characterized by their selective cytotoxicity against specific cancer cell lines while exhibiting minimal or negligible effects on non-tumorigenic cells [7]. Among them, Mpp46Aa1 (formerly PS2Aa1) belongs to the family of aerolysin-like β-pore-forming proteins and has demonstrated activity against colorectal cancer, leukemia, and other carcinoma cell lines. In recent years, protein engineering approaches, including site-directed mutagenesis and the design of bioactive peptide fragments, have been proposed to improve the efficacy, selectivity, and therapeutic potential of Mpp46Aa1-derived molecules [4,15,16].

Previous studies demonstrated that targeted modifications of specific residues in native Mpp46Aa1 can substantially alter its biological activity. For example, the G257V substitution in the 3–35 variant enhanced cytotoxicity against colorectal cancer cells, whereas the N65 variant, which contains the substitutions N92D, K175R, and S218G, exhibited increased activity against SW620, MOLT-4, and Jurkat cells [4,8]. However, because these substitutions were simultaneously present in N65, their individual contributions to the improved phenotype could not be determined. In the present study, the generation of individual reversion variants demonstrated that these residues contribute unequally to the biological activity of N65, revealing the specific role of each substitution in modulating cytotoxicity and selectivity.

Among the variants evaluated, D34N displayed the most favorable functional profile, combining low IC_50_ values in SW480 and SW620 cells with the highest selectivity index relative to the non-tumorigenic NCM460 cell line. The D34N SI of 12.08 in SW620 represents favorable discrimination relative to NCM460 within this experimental system but does not constitute a clinical therapeutic window. Direct SI comparisons across studies are limited by differences in cell models, exposure time, concentration range, and IC50 fitting [17,18]. Restoration of asparagine at this position enhanced both cytotoxic efficacy and tumor selectivity. Importantly, the reduction in cell viability was accompanied by phosphatidylserine externalization, mitochondrial membrane depolarization, and activation of initiator (caspase-9) and effector (caspases-3/7) caspases, supporting the induction of apoptosis rather than a nonspecific decrease in metabolic activity [4,19,20]. A limitation of this study is that the functional assays were evaluated at single endpoint time points (24 h for apoptosis-related assays and 72 h for metabolic activity). Therefore, the kinetics and temporal sequence of cell-death events cannot be established, and future time-course studies will be required to characterize the progression of the response induced by D34N. Kinetic studies have shown that these events can occur asynchronously and with a stimulus- and cell model-dependent sequence [21,22]. Therefore, time curves could be included in future studies to more accurately define the progression of the D34N-induced response.

The mitochondrial depolarization induced by D34N is likely a secondary event within the cytotoxic process. Mpp46Aa1 initially acts at the plasma membrane through receptor binding, oligomerization, and pore formation, leading to membrane permeabilization [23]. This disruption of cellular homeostasis may subsequently compromise mitochondrial function and trigger activation of caspase-9 and caspases-3/7, resulting in a response consistent with the intrinsic apoptotic pathway previously described for Mpp46Aa1 [4,7]. The concordance among these events is consistent with observations reported for the N65 and 3–35 variants, as well as for other parasporins whose apoptotic effects depend on cell type, protein concentration, and exposure time [4,7,24,25].

From a structural perspective, the enhanced activity of D34N may be associated with localized physicochemical changes around residue 34 of the activated protein, corresponding to residue 92 in the precursor sequence [4]. This reversion replaces the negatively charged carboxylate group of aspartate with the neutral carboxamide group of asparagine [26,27]. Although both residues participate in hydrogen bonding, asparagine can act as both a hydrogen bond donor and acceptor, potentially altering local interaction geometry and electrostatic distribution, thereby affecting local structural organization [28]. Consistent with this interpretation, electrostatic surface analysis suggested a localized reduction in negative potential surrounding residue 34 without detectable alterations in the global protein fold. The low RMSD values (0.001–0.002 Å) indicate near-identical global geometric similarity among static models but do not demonstrate dynamic stability or exclude relevant local changes. Therefore, they should not be interpreted as evidence of altered global conformation, dynamic stability, or functionally relevant structural differences. Rather, these values may partly reflect the limitations of comparative in silico modeling, which may not capture local conformational flexibility, protein–membrane interactions, oligomerization, or receptor-dependent effects.

This observation may be functionally relevant because Domain I of Mpp46Aa1 has been proposed to mediate target-cell recognition, whereas Domains II and III participate in oligomerization and pore formation, respectively [6,9]. Previous molecular simulations of the Mpp46Aa1–GPI–CD59 complex identified Asn92 among the residues maintaining contact with CD59 in two out of three independent simulations, suggesting that this residue may contribute to receptor recognition or stabilization of protein–membrane interactions [4]. The structural analysis is presented only as a hypothesis-generating observation. We also explicitly state that direct binding and oligomerization assays will be required to determine whether D34N alters membrane or coreceptor recognition. Nevertheless, GPI-anchored proteins are thought to function primarily as co-receptors facilitating parasporin binding and oligomerization rather than determining cellular specificity [11,29]. Therefore, the D34N substitution may improve the positioning or interaction efficiency of Mpp46Aa1 at the cell surface, although it should not be considered the sole determinant of selectivity.

The distinct responses observed among SW480, SW620, and NCM460 cells indicate that susceptibility to Mpp46Aa1 variants depends not only on protein sequence but also on the molecular context of the target cell, including membrane composition and receptor availability, which may explain the differences in IC_50_ values and selectivity indices [4,8]. Mpp46Aa1 activity requires localization within lipid rafts and the formation of oligomeric complexes, processes influenced by membrane proteins, cholesterol content, and other bilayer components [30]. Variations in membrane organization, glycosylation patterns, or receptor distribution may therefore affect pore formation efficiency, although these parameters were not evaluated in the present study. Cellular responses may also depend on membrane repair mechanisms and the status of apoptotic pathways, factors known to influence sensitivity to pore-forming proteins [4,7,31].

The interpretation of these findings should consider that all experiments were performed using two-dimensional cell culture models under controlled exposure conditions. Future studies should experimentally characterize membrane binding, oligomerization, and pore formation by Mpp46Aa1 variants. Furthermore, emerging strategies, including the development of functional peptide fragments and the incorporation of Mpp46Aa1 into nanoscale delivery systems, suggest that engineering both the protein and its delivery platform may simultaneously improve stability, bioavailability, efficacy, and selectivity [15,21].

## 5. Conclusions

The D34N reversion variant exhibited the most favorable biological profile among the proteins evaluated, combining enhanced cytotoxicity against SW480 and SW620 colorectal cancer cells with superior selectivity relative to NCM460 cells. The reduction in cell viability was associated with phosphatidylserine externalization, mitochondrial membrane depolarization, and activation of caspase-9 and caspases-3/7, supporting the induction of apoptosis through the intrinsic mitochondrial pathway.

Structural analyses demonstrated that the D34N substitution induced localized changes in molecular interactions and electrostatic surface properties without altering the overall three-dimensional architecture of the protein. These findings suggest that restoration of asparagine at residue 34 contributes to target-cell recognition and enhances the cytotoxic efficiency of Mpp46Aa1 through local physicochemical effects rather than global conformational changes.

Collectively, these results identify D34N as a promising engineered Mpp46Aa1 variant for the rational design of selective antitumor proteins. Further studies employing three-dimensional tumor models, receptor-binding analyses, and preclinical systems will be required to validate its therapeutic potential and to elucidate the molecular determinants underlying its enhanced activity.

## Figures and Tables

**Table 1:** Estimated IC_50_ values of recombinant Mpp46Aa1 proteins in SW480, SW620, and NCM460 cell lines following 72 h of treatment. IC_50_ values were calculated from dose–response curves generated using the AlamarBlue cell viability assay. Values are expressed as IC_50_ in μg/mL, followed in parentheses by the 95% confidence interval (95% CI).

	Celular Lines		
Parasporin	SW480 IC_50_	SW620 IC_50_	NCM460 IC_50_
R117K	13.76 (9.31–21.16)	5.81 (4.65–7.28)	18.31 (11.15–32.86)
G160S	2.14 (1.03–4.20)	1.57 (0.98–2.42)	6.04 (4.11–8.98)
D34N	3.86 (2.86–5.21)	1.52 (0.81–2.64)	18.36 (15.26–22.33)
N65	9.13 (7.10–12.34)	4.71 (3.16–7.01)	14.39 (10.76–19.67)
Mpp46Aa1	7.53 (4.49–13.07)	4.63 (2.68–8.03)	15.96 (8.78–32.74)

**Table 2:** Selectivity index (SI) of recombinant Mpp46Aa1 proteins against colorectal cancer cell lines. The SI was calculated from the ratio of IC_50_ values obtained in NCM460 cells relative to those in SW480 and SW620 cells. Higher SI values indicate greater selectivity toward cancer cells and a more favorable therapeutic profile.

	Celular Lines	
Parasporin	SW480	SW620
R117K	1.33	3.15
G160S	2.83	3.84
D34N	4.75	12.06
N65	1.55	3.06
Mpp46Aa1	2.12	3.45

**Table 3:** Root mean square deviation (RMSD) values obtained from the structural alignment of Mpp46Aa1 variants. Structural superposition was performed using PyMOL, and RMSD values are reported in ångströms (Å). RMSD was used to quantify the degree of structural similarity between variants and to assess whether the observed functional differences were associated with detectable conformational changes.

Structural	Aligned Atoms	RMSD
N65/2ZTB	1461	0.002 Å
D34N/2ZTB	1464	0.002 Å
D34N/N65	1631	0.001 Å

## Data Availability

Supporting data is available upon reasonable request from the relevant author.

## Supplementary Materials

The following supporting information can be downloaded at: https://www.mdpi.com/article/10.3390/cancers18182980/s1. Table S1: Values correspond to three independent biological replicates (*n* = 3), each obtained from the mean of three technical replicates. Luminescence was recorded as relative luminescence units (RLU), and no additional normalization was performed. Data are presented as mean, standard deviation (SD), and 95% confidence interval.
