# Supplementary material for: Structure–Function Analysis of Individual Reversion Variants of the Engineered Mpp46Aa1 (PS2Aa1) N65 Protein
Source: Cancers (Basel). 2026 Sep 15;18(18):2980. doi: 10.3390/cancers18182980 (PMC13605735; doi:10.3390/cancers18182980)

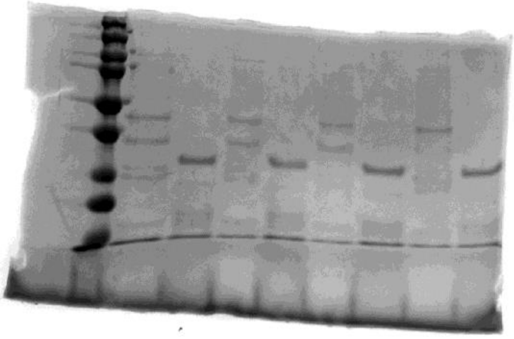

Supplement: Supplementary file 1 [file cancers-18-02980-s001.zip › cancers-4543538-original-images.pdf]
