# Supplementary material for: Structure–Function Analysis of Individual Reversion Variants of the Engineered Mpp46Aa1 (PS2Aa1) N65 Protein
Source: Cancers (Basel). 2026 Sep 15;18(18):2980. doi: 10.3390/cancers18182980 (PMC13605735; doi:10.3390/cancers18182980)
Supplement: Supplementary file 1 [file cancers-18-02980-s001.zip › cancers-4543538-supplementary.pdf]

Values correspond to three independent biological replicates (n = 3), each obtained from the mean of three technical replicates.
Luminescence was recorded as relative luminescence units (RLU), and no additional normalization was performed.
Data are presented as mean, standard deviation (SD), and 95% confidence interval.

SW480					
Caspases 3/7					
Treatment	Technical replicate 1	Technical replicate 2	Technical replicate 3	Biological replicate	Mean RLU
C-	5608	5614	5617	5613	5616
	5612	5619	5623	5618	
	5611	5617	5620	5616	
D34N	12618	12624	12627	12623	12620
	12615	12621	12624	12620	
	12613	12619	12622	12618	
N65	12118	12124	12127	12123	12118
	12108	12114	12117	12113	
	12111	12118	12122	12117	
Mpp46Aa1	11218	11224	11227	11223	11230
	11227	11234	11238	11233	
	11229	11236	11240	11235	

SW480					
Caspase 9					
Treatment	Technical replicate 1	Technical replicate 2	Technical replicate 3	Biological replicate	Mean RLU
C-	5208	5214	5217	5213	5213
	5209	5212	5218	5213	
	5207	5215	5217	5213	
D34N	20288	20277	20284	20283	20242
	20147	20155	20157	20153	
	20284	20291	20295	20290	
N65	18338	18344	18347	18343	18343
	18327	18334	18338	18333	
	18348	18354	18357	18353	
Mpp46Aa1	13334	13339	13341	13338	13339
	13335	13337	13341	13338	
	13336	13343	13347	13342	

SW620					
Caspases 3/7					
Treatment	Technical replicate 1	Technical replicate 2	Technical replicate 3	Biological replicate	Mean RLU
C-	3878	3884	3887	3883	3877
	3888	3894	3897	3893	
	3851	3857	3860	3856	
D34N	11598	11604	11607	11603	11695
	11697	11705	11707	11703	
	11774	11781	11785	11780	
N65	10081	10087	10090	10086	10090
	10088	10094	10097	10093	
	10084	10091	10095	10090	
Mpp46Aa1	8983	8989	8992	8988	8985
	8988	8994	8997	8993	
	8968	8974	8977	8973	

SW620					
Caspase 9					
Treatment	Technical replicate 1	Technical replicate 2	Technical replicate 3	Biological replicate	Mean RLU
C-	3788	3794	3797	3793	3780
	3758	3764	3767	3763	
	3778	3784	3787	3783	
D34N	22538	22544	22547	22543	22541
	22517	22524	22528	22523	
	22550	22557	22561	22556	
N65	18948	18954	18957	18953	18956
	18968	18974	18977	18973	
	18938	18944	18947	18943	
Mpp46Aa1	9578	9584	9587	9583	9585
	9588	9594	9597	9593	
	9574	9580	9583	9579	

NCM460					
Caspases 3/7					
Treatment	Technical replicate 1	Technical replicate 2	Technical replicate 3	Biological replicate	Mean RLU
C-	3261	3267	3270	3266	3189
	3135	3141	3144	3140	
	3154	3161	3165	3160	
D34N	3258	3264	3267	3263	3196
	3168	3174	3177	3173	
	3148	3154	3157	3153	
N65	3128	3134	3137	3133	3098
	3018	3024	3027	3023	
	3133	3139	3142	3138	
Mpp46Aa1	3015	3021	3024	3020	3084
	3108	3114	3117	3113	
	3114	3120	3123	3119	

NCM460					
Caspase 9					
Treatment	Technical replicate 1	Technical replicate 2	Technical replicate 3	Biological replicate	Mean RLU
C-	3261	3267	3270	3266	3155
	3135	3141	3144	3140	
	3055	3061	3064	3060	
D34N	3258	3264	3267	3263	3163
	3068	3074	3077	3073	
	3148	3154	3157	3153	
N65	3028	3034	3037	3033	3065
	3018	3024	3027	3023	
	3133	3139	3142	3138	
Mpp46Aa1	3015	3021	3024	3020	3051
	3108	3114	3117	3113	
	3014	3020	3023	3019	
